# Inherited Breast Cancer in Nigerian Women

**DOI:** 10.1200/JCO.2018.78.3977

**Published:** 2018-08-21

**Authors:** Yonglan Zheng, Tom Walsh, Suleyman Gulsuner, Silvia Casadei, Ming K. Lee, Temidayo O. Ogundiran, Adeyinka Ademola, Adeyinka G. Falusi, Clement A. Adebamowo, Abideen O. Oluwasola, Adewumi Adeoye, Abayomi Odetunde, Chinedum P. Babalola, Oladosu A. Ojengbede, Stella Odedina, Imaria Anetor, Shengfeng Wang, Dezheng Huo, Toshio F. Yoshimatsu, Jing Zhang, Gabriela E.S. Felix, Mary-Claire King, Olufunmilayo I. Olopade

**Affiliations:** Yonglan Zheng, Shengfeng Wang, Dezheng Huo, Toshio F. Yoshimatsu, Jing Zhang, Gabriela E.S. Felix, and Olufunmilayo I. Olopade, The University of Chicago, Chicago, IL; Tom Walsh, Suleyman Gulsuner, Silvia Casadei, Ming K. Lee, and Mary-Claire King, University of Washington, Seattle, WA; Temidayo O. Ogundiran, Adeyinka Ademola, Adeyinka G. Falusi, Abideen O. Oluwasola, Adewumi Adeoye, Abayomi Odetunde, Chinedum P. Babalola, Oladosu A. Ojengbede, Stella Odedina, Imaria Anetor, University of Ibadan; Clement A. Adebamowo, Centre for Bioethics and Research, Ibadan, Oyo, Nigeria, and University of Maryland School of Medicine, Baltimore, MD; and Gabriela E.S. Felix, Fundação Oswaldo Cruz and Universidade Federal da Bahia, Bahia, Brazil.

## Abstract

**Purpose:**

Among Nigerian women, breast cancer is diagnosed at later stages, is more frequently triple-negative disease, and is far more frequently fatal than in Europe or the United States. We evaluated the contribution of an inherited predisposition to breast cancer in this population.

**Patients and Methods:**

Cases were 1,136 women with invasive breast cancer (mean age at diagnosis, 47.5 ± 11.5 years) ascertained in Ibadan, Nigeria. Patients were selected regardless of age at diagnosis, family history, or prior genetic testing. Controls were 997 women without cancer (mean age at interview, 47.0 ± 12.4 years) from the same communities. BROCA panel sequencing was used to identify loss-of-function mutations in known and candidate breast cancer genes.

**Results:**

Of 577 patients with information on tumor stage, 86.1% (497) were diagnosed at stage III (241) or IV (256). Of 290 patients with information on tumor hormone receptor status and human epidermal growth factor receptor 2, 45.9% (133) had triple-negative breast cancer. Among all cases, 14.7% (167 of 1,136) carried a loss-of-function mutation in a breast cancer gene: 7.0% in *BRCA1*, 4.1% in *BRCA2*, 1.0% in *PALB2*, 0.4% in *TP53*, and 2.1% in any of 10 other genes. Odds ratios were 23.4 (95% CI, 7.4 to 73.9) for *BRCA1* and 10.3 (95% CI, 3.7 to 28.5) for *BRCA2*. Risks were also significantly associated with *PALB2* (11 cases, zero controls; *P* = .002) and *TP53* (five cases, zero controls; *P* = .036). Compared with other patients, *BRCA1* mutation carriers were younger (*P* < .001) and more likely to have triple-negative breast cancer (*P* = .028).

**Conclusion:**

Among Nigerian women, one in eight cases of invasive breast cancer is a result of inherited mutations in *BRCA1*, *BRCA2*, *PALB2*, or *TP53*, and breast cancer risks associated with these genes are extremely high. Given limited resources, prevention and early detection services should be especially focused on these highest-risk women.

## INTRODUCTION

Among Nigerian women, breast cancer generally is diagnosed at an advanced stage, and survival is very poor.^1,2^ In addition, Nigerian women are diagnosed more frequently with triple-negative breast cancer (TNBC) than patients of European ancestry.^3^ Breast cancer incidence in this population historically has been low but is now increasing.^4^ Given limited resources for population screening by mammography, the identification of women at especially high risk of breast cancer is useful to focus screening efforts particularly for them.

The goals of the project reported herein were to determine the proportion of breast cancer as a result of inherited disease among Nigerian women, the breast cancer genes that most frequently harbor pathogenic mutations in this population, and the increases in breast cancer risks associated with mutations in these genes. Some Nigerian patients with breast cancer were previously screened for a few specific alleles of *BRCA1* and *BRCA2*,^5-8^ but no African population has been evaluated for all known and candidate breast cancer genes. Recent advances in genomic technology now enable simultaneous sequencing of all such genes.^9,10^ In addition, community engagement has led to study enrollment of unaffected women of the same ages and ethnic and socioeconomic backgrounds as the cases, which enables risk estimates on the basis of appropriate controls.

## PATIENTS AND METHODS

### Study Participants

The Nigerian Breast Cancer Study is a case-control study with enrollment between March 1998 and 2014. The study setting and design have been described in detail elsewhere.^11-14^ Briefly, all cases were diagnosed and histologically confirmed as invasive breast cancer by pathologists at the University College Hospital in Ibadan, Nigeria, a tertiary hospital that serves southwestern Nigeria. All cases were at least 18 years old and were included regardless of age at diagnosis, family history, or previous genetic testing. Histologic diagnosis was based on evaluation of hematoxylin and eosin–stained slides. Tumors of a subset of the patients also were evaluated by immunohistochemistry for estrogen receptor (ER), progesterone receptor (PR), and human epidermal growth factor receptor 2 (HER2). Controls were recruited from hospital general outpatient clinics and communities that represent the diversity of ethnicities and socioeconomic status of University College Hospital patients with cancer. Institutional review boards of The University of Chicago, the University of Ibadan, and the University of Washington approved the study. All participants in this study provided written informed consent. On the basis of institutional review board review, genetic testing results from this study were considered research and were not returned to the study participants. The study enrolled 1,136 cases and 997 controls (386 hospital based and 611 community based).

### Genomics

Genomic DNA was sequenced using the BROCA gene panel,^9,15^ which enabled the identification of all classes of mutations (point mutations, small insertions and deletions, and genomic deletions and duplications) in coding sequence, introns, untranslated regulatory regions, and 10 kb preceding and after the transcription start and stop sites of each gene. Genes on the BROCA panel included established breast cancer genes of both high and moderate penetrance and genes that have been suggested as candidate for breast cancer predisposition. Strength of evidence for these candidate genes varies. Genes included were *BRCA1*, *BRCA2*, *ATM*, *ATR*, *BAP1*, *BARD1*, *BRIP1*, *CDH1*, *CHEK1*, *CHEK2*, *FAM175A*, *FANCM*, *GEN1*, *MRE11A*, *NBN*, *PALB2*, *PTEN*, *RAD51B*, *RAD51C*, *RAD51D*, *RECQL*, *RINT1*, *SLX4*, *TP53*, and *XRCC2*. Paired-end reads with median 250× coverage were aligned to the human genome reference hg19. Alignment, base quality calibration, and variant identification were carried out as previously described.^9,15^ Genomic deletions and duplications were called by our in-house approach.^16^ Interpretations of possible enhancer and splice mutations were based on in silico programs, as previously described; on published experimental results; or on experimental results in our laboratory. Although RNA was not available from the Nigerian participants, transcriptional effects of splice variants that had appeared in other studies in our laboratory could be tested. Potential splice or enhancer mutations were included only if shown experimentally (by us or others) to alter splicing. In-frame deletions, either in DNA or RNA, were included only if a critical domain was deleted. Only mutations that led to loss of gene function or were experimentally demonstrated to damage gene function were included in subsequent analyses. Statistical analysis of categorical variables was based on two-tailed χ^2^ tests, with Pearson continuity correction, or by Fisher’s exact tests, as appropriate. Continuous variables were compared by *t* tests or by Wilcoxon rank sum (Mann-Whitney *U*) tests if not normally distributed. Odds ratios (ORs) and 95% CIs were calculated by established methods.

## RESULTS

Demographic and clinical characteristics of the patients and controls are listed in Table 1. Mean age at diagnosis of breast cancer cases was 47.54 ± 11.47 years, and mean age at interview of controls was 46.99 ± 12.44 years. Six percent of patients and 2% of controls reported a family history of breast cancer; for many participants, no information on family medical history was available. Of patients with information on tumor stage, 86.1% (497 of 577) were diagnosed at stage III (241 of 577) or stage IV (246 of 577). Of patients with information on tumor ER, PR, and HER2 status, 45.9% (133 of 290) had TNBC.

**Table 1.**
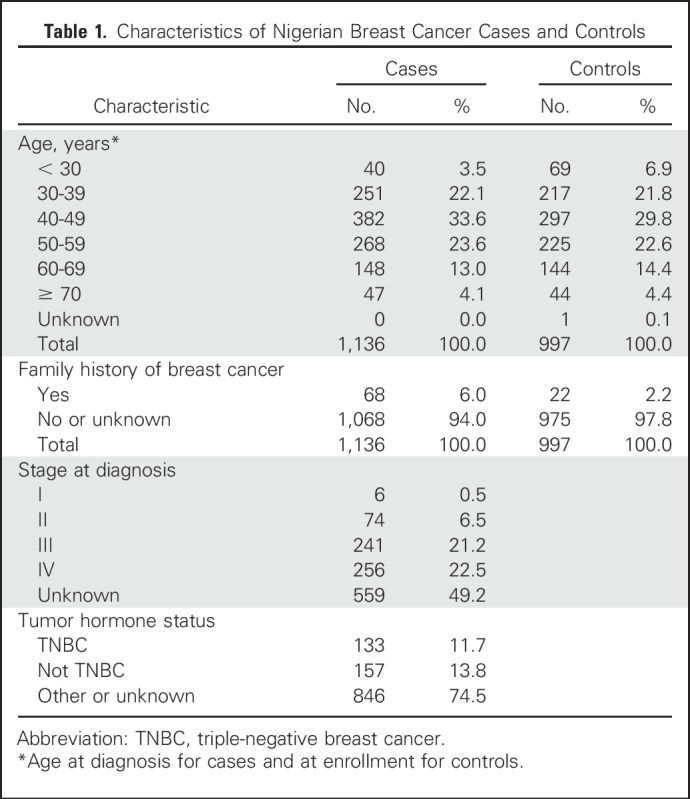
Characteristics of Nigerian Breast Cancer Cases and Controls

**Fig 1.**
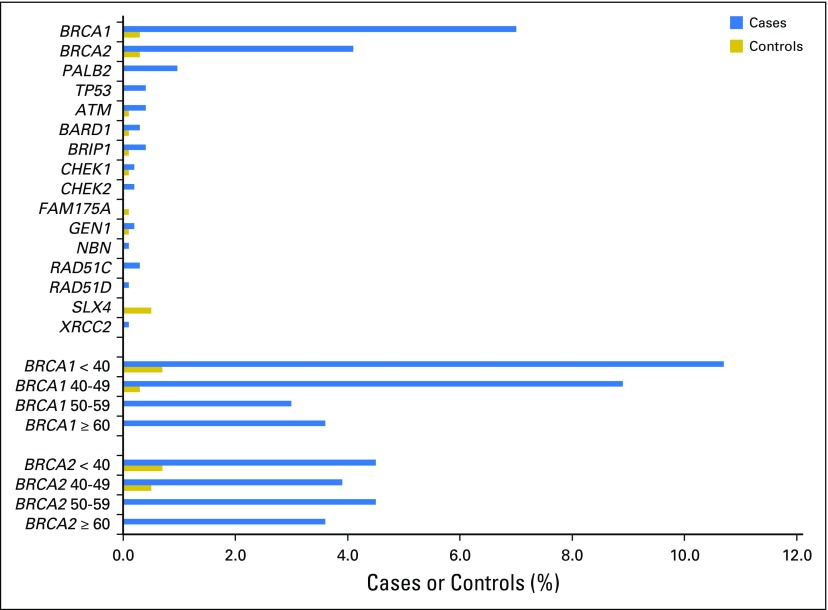
Damaging mutations in known and candidate breast cancer genes in Nigerian women. The graph indicates the percentages of 1,136 cases and 997 controls identified as carriers of a damaging mutation in a known or candidate breast cancer gene on the basis of sequencing with the BROCA gene panel. Differences between cases and controls were significant for *BRCA1*, *BRCA2*, *PALB2*, and *TP53* (see Results). Graphs at the bottom of the figure represent percentages of mutation carriers for *BRCA1* and *BRCA2* for cases and controls stratified by age at diagnosis (for cases) or age at interview (for controls).

Among all patients with breast cancer, 14.7% (167 of 1,136) carried an unambiguously damaging mutation in a breast cancer gene, whereas among controls, 1.8% (18 of 997) carried such a mutation (Table 2; Fig 1). Six mutations in cases and two mutations in controls were large deletions (Data Supplement). The gene that contributed most to risk was *BRCA1*, both because 7.0% of patients (80 of 1,136) harbored a damaging mutation in *BRCA1* and because the increase in breast cancer risk associated with *BRCA1* mutation was extremely high (OR, 23.40; 95% CI, 7.41 to 73.88; *P* < .001). The gene that contributed the next most severely to risk was *BRCA2*, with 4.1% of patients harboring a damaging mutation and a significantly increased risk (OR, 10.76; 95% CI, 3.86 to 29.99; *P* < .001).

**Table 2.**
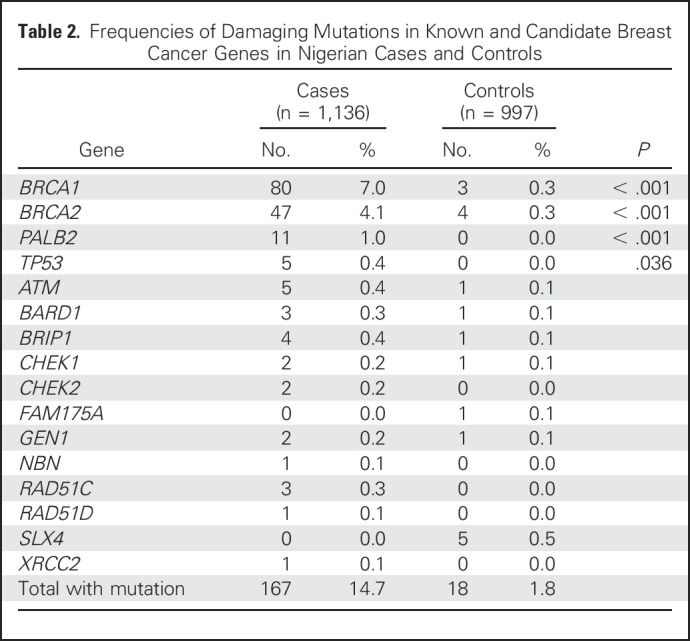
Frequencies of Damaging Mutations in Known and Candidate Breast Cancer Genes in Nigerian Cases and Controls

*PALB2* and *TP53* also were associated with significant increases in breast cancer risk, with 11 patients and zero controls carrying a damaging mutation in *PALB2* (*P* < .001), and four patients and zero controls carrying a damaging mutation in *TP53* (*P* = .036). Of the *TP53* mutations, one was a frameshift and four were missenses with evidence for partial loss of function (Data Supplement). Insofar as could be determined, none of the patients with *TP53* mutations had relatives with other Li-Fraumeni syndrome cancers.

Ten other genes—*ATM*, *BARD1*, *BRIP1*, *CHEK1*, *CHEK2*, *GEN1*, *NBN*, *RAD51C*, *RAD51D*, and *XRCC2*—each harbored a damaging mutation in one or more patients (Table 2). One patient carried damaging mutations in two genes, *BRCA1* and *BRIP1*. *CHEK2* played a far more minor role in this population than in European populations; no common Nigerian mutations were found in *CHEK2* (Data Supplement). Two genes, *FAM175A* and *SLX4*, harbored damaging mutations in controls but not in cases (Table 2), consistent with other observations that mutations in these two genes do not predispose to breast cancer.^17^ Mutations in the known moderate-risk genes were too rare to yield meaningful gene-specific risk estimates, and the evidence for causality among these genes varies too widely to pool risk estimates. Results from the Nigerian population do not provide strong evidence for or against association with breast cancer for these genes. No cases or controls carried damaging mutations in any of the other genes on the BROCA panel.

Age at diagnosis was significantly younger for *BRCA1* mutation carriers (42.63 ± 10.14 years) than for other patients (47.90 ± 11.49 years; *P* < .001) and for *TP53* mutation carriers (32.80 ± 9.26 years) than for other patients (47.60 ± 11.44 years; *P* = .023). Age at diagnosis was not associated with mutations in any other gene.

Both allelic heterogeneity and founder mutations played a role in inherited breast cancer (Data Supplement). Allelic heterogeneity among the patients was reflected in the appearance of 105 different mutations in 14 genes. On the other hand, approximately one half of patients (52.7% [88 of 167]) carried a mutation present in at least one other case. Of the most common mutations among the patients, *BRCA1* p.M1775R is of particular historical interest because it was the first *BRCA1* mutation identified in an African American family.^18^

TNBC was significantly associated with mutations in *BRCA1* (Data Supplement). Among patients with tumors known to be TNBC, 8.3% (11 of 133) carried a *BRCA1* mutation, whereas among patients with tumors known to be positive for ER, PR, or HER2, 2.5% (four of 157) carried a *BRCA1* mutation (OR, 3.45; 95% CI, 1.07 to 11.10; *P* = .028). Patients with TNBC were slightly, but not significantly, older at diagnosis than patients with non-TNBC tumors (49.39 ± 12.15 *v* 47.40 ± 11.67 years; *P* = .16); the exclusion of *BRCA1* mutation carriers from these calculations did not change the result. TNBC was not associated with tumor stage (*P* = .96).

## DISCUSSION

Results of genomic analysis of Nigerian women with breast cancer and age- and community-matched controls suggest several themes important to clinical translation of cancer genetics both in Africa and in general. First, for Nigerian women, *BRCA1* and *BRCA2* have a major effect on breast cancer incidence both because the ORs for *BRCA1* and *BRCA2* are extremely high (> 20 for *BRCA1*; > 10 for *BRCA2*) and because 11% of patients carry a damaging mutation in one of these genes (7.0% in *BRCA1*; 4.1% in *BRCA2*). These carrier frequencies are much higher than those reported from population-based screening of African American patients with breast cancer on the basis of earlier mutation detection methods^19^ and are more comparable to carrier frequencies among African American women referred for clinical genetic testing.^20,21^ Mutations in *PALB2* and *TP53* also confer a significantly elevated risk, although mutations in these genes were less frequent, and risks were not estimable because no mutations appeared in controls.

 The role of inherited predisposition to breast cancer risk in Nigeria is important to understand in the context of the currently increasing risks of breast cancer among Nigerian women.^4^ As elsewhere, it is likely that breast cancer incidence is increasing among Nigerian women primarily as a result of better nutrition in young girls, which leads to earlier menarche, and better education of young women, which leads to later age at first pregnancy. We note that gene-environment interaction thus plays an important role in this increase. Studies in western countries indicate that among *BRCA1* and *BRCA2* mutation carriers, breast cancer risks, age for age, are significantly higher among women born more recently than among women born earlier, even with the same mutations in the same families.^22,23^ The causes of the increase in risk by birth cohort are not genetic but are changes in the same aforementioned features of reproductive history. In other words, risks of breast cancer to *BRCA1* or *BRCA2* mutation carriers is context dependent and varies by geography and the social environment in which the mutation carrier lives.^24^

Second, most of the well-documented high prevalence of TNBC in this population remains unexplained.^3^ TNBC is significantly associated with *BRCA1* carrier status among Nigerian patients, as elsewhere.^25-27^ However, > 90% of the Nigerian patients with TNBC studied had no mutation in *BRCA1*. The biologic basis of TNBC in African and African American women remains a critical unanswered question.

Third, genetic screening for patients with breast cancer is useful only if it is comprehensive, with full sequencing of all breast cancer genes. The importance of both allelic heterogeneity and founder mutations among Nigerian patients echoes the pattern seen in Europe^28^ and explains the previous observation in this population that recurrent mutations identified in a small discovery series of patients were not good predictors of mutations in a second series from the same population.^8^ To screen only for recurrent mutations, even in several genes, would lead to missing the mutations of > 50% of women. Comprehensive sequencing for breast cancer genes is now feasible on a large scale and could be deployed to improve access to personalized screening by mammography and other detection methods for women who need it most.

Finally, this project offered the opportunity to evaluate patients with breast cancer and controls ascertained regardless of age at diagnosis or family history or previous genetic testing. To carry out such a survey in high-income countries is challenging because many patients, particularly those with young-onset diagnoses or severe family histories, have been tested commercially. These problems have been addressed by sequencing large series of patients from clinical trials,^29^ but such series do not include controls. The Nigerian series was designed to minimize ascertainment biases and to include controls from the same geographic locales as the patients. Results revealed very high risks for carriers of mutations in *BRCA1* and *BRCA2* and a high prevalence of mutation carriers among these patients.

The principal limitation of this study was that histopathologic features of tumors, including stage and hormonal status, were available for only a minority of patients. Lack of this information constrained efforts to characterize the high prevalence of TNBC in this population. These issues reflect resource-limited settings generally and support the importance of developing independent, inexpensive approaches to the identification of high-risk women.

In conclusion, we suggest that genomic sequencing to identify women at extremely high risk of breast cancer could be a highly innovative approach to tailored risk management and life-saving interventions. An urgent need exists to address widening global disparities in breast cancer mortality that disproportionately affect women of African ancestry both in Africa and throughout the diaspora. In the United States, African American women have the highest breast cancer mortality rate.^30^ Given that breast cancer is more frequently TNBC among both African American and Nigerian women than among other populations^3^ and given the very young ages at diagnosis in the Nigerian population, a focus on risk management in genetically high-risk women could substantially reduce premature mortality as a result of breast cancer in Nigeria.

 Application of genomic technology to breast cancer risk stratification is consistent with the WHO Human Genomics in Global Health Initiative^31^ and with the United Nations Sustainable Development Goals for 2015 to 2030.^32^ It may seem paradoxical to apply the most recent technology in severely resource-limited settings, but the solution fits the problem well. More than 20 years after the first extended family of African ancestry with a *BRCA1* mutation was published,^18^ the critical genes and classes of mutations responsible for the high risk of inherited breast cancer among Nigerian women are now clear. On the basis of our results, the critical genes for inherited breast cancer in this population are *BRCA1*, *BRCA2*, *PALB2*, and *TP53*, and the critical mutations in these genes are those that lead to loss of function. Nigeria now has data to prioritize the integration of genetic testing into its cancer control plan. Women with an extremely high risk of breast cancer as a result of mutations in these genes can be identified inexpensively and unambiguously and offered interventions to reduce cancer risk. In Nigeria, women with cancer-predisposing mutations in these genes comprise 12% to 13% of all patients with breast cancer. One half of sisters and daughters will carry the mutation of the index patient. The current results indicate that approximately one in 150 unaffected young women from the general population also will carry such a mutation. If these women at very high risk can be identified either through their relatives with breast cancer or in the general population, resources can be focused particularly on their behalf. For as-yet unaffected women at high genetic risk, these resources would be intensive surveillance for early detection of breast cancer and, after childbearing is completed, the possibility of preventive salpingo-oophorectomy.^33^ Integrated population screening for cancer for all women is the goal, but focused outreach to women at extremely high risk represents an especially efficient use of resources and an attainable evidence-based global health approach.
